# Identification of candidate diagnostic serum biomarkers for Kawasaki disease using proteomic analysis

**DOI:** 10.1038/srep43732

**Published:** 2017-03-06

**Authors:** Yayoi Kimura, Masakatsu Yanagimachi, Yoko Ino, Mao Aketagawa, Michie Matsuo, Akiko Okayama, Hiroyuki Shimizu, Kunihiro Oba, Ichiro Morioka, Tomoyuki Imagawa, Tetsuji Kaneko, Shumpei Yokota, Hisashi Hirano, Masaaki Mori

**Affiliations:** 1 Advanced Medical Research Center, Yokohama City University, Yokohama, Japan; 2 Department of Pediatrics, Yokohama City University School of Medicine, Yokohama, Japan; 3 Department of Pediatrics, Tokyo Medical and Dental University, Tokyo, Japan; 4 Department of Pediatrics, Yokohama City University Medical Center, Yokohama, Japan; 5 Department of Pediatrics, Showa General Hospital, Tokyo, Japan; 6 Department of Pediatrics, Kobe University Graduate School of Medicine, Kobe, Japan; 7 Department of Infectious Disease & Immunology, Kanagawa Children’s Medical Center, Yokohama, Japan; 8 Teikyo Academic Research Center, Teikyo University, Tokyo, Japan; 9 Department of Lifetime Clinical Immunology, Graduate School of Medical and Dental Sciences, Tokyo Medical and Dental University, Tokyo, Japan.

## Abstract

Kawasaki disease (KD) is a systemic vasculitis and childhood febrile disease that can lead to cardiovascular complications. The diagnosis of KD depends on its clinical features, and thus it is sometimes difficult to make a definitive diagnosis. In order to identify diagnostic serum biomarkers for KD, we explored serum KD-related proteins, which differentially expressed during the acute and recovery phases of two patients by mass spectrometry (MS). We identified a total of 1,879 proteins by MS-based proteomic analysis. The levels of three of these proteins, namely lipopolysaccharide-binding protein (LBP), leucine-rich alpha-2-glycoprotein (LRG1), and angiotensinogen (AGT), were higher in acute phase patients. In contrast, the level of retinol-binding protein 4 (RBP4) was decreased. To confirm the usefulness of these proteins as biomarkers, we analyzed a total of 270 samples, including those collected from 55 patients with acute phase KD, by using western blot analysis and microarray enzyme-linked immunosorbent assays (ELISAs). Over the course of this experiment, we determined that the expression level of these proteins changes specifically in the acute phase of KD, rather than the recovery phase of KD or other febrile illness. Thus, LRG1 could be used as biomarkers to facilitate KD diagnosis based on clinical features.

Kawasaki disease (KD) is a systemic vasculitic disease whose etiology remains unknown. Moreover, KD can lead to cardiovascular complications such as coronary artery lesions (CALs)^1^,^2^. KD is the most frequent cause of childhood ischemic heart disease. The disease was first reported by Dr. T. Kawasaki, a Japanese pediatrician, in 1967^3^, and took more than ten years to become widely known as a new disease entity. However, it is recognized today as the most frequent form of childhood vasculitis, apart from Henoch-Schönlein purpura^4^.

Symptoms of KD during the acute phase characteristically overlap in time, and disease manifestations are completed by the development of a fever refractory to antibiotic treatment, acute nonpurulent cervical lymphadenopathy, hyperemia of the bulbar conjunctiva, strawberry tongue, redness and cracking of the lips, hard edema of the hands and feet, and angiitis symptoms such as redness of finger tips by day 3 to day 7 after the onset. Moreover, in cases accompanied by development of CALs, echocardiography shows increased brightness of coronary arteries around day 10, and dilatation and aneurysm formation of the coronary arteries are detected between day 10 and day 14. Delaying definitive diagnosis of KD leads to development of CALs. However, there are no specific biomarkers for KD definitive diagnosis in clinical settings. The diagnosis of KD depends on its clinical features^2^. Therefore, it is often difficult to decide on the intervention for KD, especially in patients with incomplete clinical characteristics (incomplete KD), in the absence of a definitive diagnostic test^5^. Diagnostic methods based on biomarkers are necessary to assist physicians in diagnosing KD.

According to the 22^nd^ national survey on KD in Japan^6^, there were more than 10,000 onset cases per year after 2005. KD cases have recently been increasing in number more rapidly. The morbidity is currently at 264.8 per 100,000 in the population of 0 to 4 year-olds in 2012. CALs used to develop in 25–30% of cases during the acute phase of KD, but since the introduction of intravenous immunoglobulin (IVIG) in the latter half of the 1980s, the onset rate of CALs has decreased to 9.3% at the acute phase, and 2.8% at the recovery phase^6^. The mortality was as high as 1% before 1974, but has decreased to 0.01% at present in Japan, probably because of intervention with IVIG^7^,^8^. Plasma exchange (PE) and anti tumor necrosis factor (TNF)-α therapy were also a safe and effective prophylactic measure against CALs in children with KD intractable to IVIG therapy^9^,^10^. Thus, safe and efficient therapeutics have been established for treating KD patients.

The pathogenesis of KD still remains obscure even almost 50 years after the disease’s discovery, although it may be summarized as abnormal activation of the immune system and panangiitis. However, the nature of KD therapeutics suggests that the serum may include a factor related to KD and CALs. Therefore, comprehensive analysis of the serum proteome of patients may be a key step in identifying candidate protein biomarkers that contribute to KD diagnosis and to the elucidation of KD pathogenesis. Serum samples are often used for biomarker discovery research. Previously, Zhang *et al*. performed two-dimensional electrophoresis (2-DE)-based analysis to compare the serum proteomes of normal children and KD patients by identifying differentially expressed proteins before and after IVIG therapy^11^. Although 2-DE is a fundamental technology for quantitative proteomics, it cannot characterize all of the protein components in serum. Recently, mass spectrometry (MS) has emerged as a powerful tool for discovering candidate protein biomarkers in proteomic studies, although cytokines and peptide hormones, which are very low-abundance proteins, are difficult to identify by MS.

In this study, we tried to comprehensively compare serum proteins from acute and recovery phase KD patients, using MS-based proteomic analysis for the identification of differentially expressed proteins. We subsequently validated KD-related proteins using specific antibodies. As a result, we identified four KD-related proteins, lipopolysaccharide-binding protein (LBP), leucine-rich alpha-2-glycoprotein (LRG1), angiotensinogen (AGT), and retinol-binding protein 4 (RBP4). These proteins are potentially useful for establishing a new diagnostic method to facilitate KD diagnosis based on clinical features.

## Results

### Identification of KD-related proteins using paired sera collected during the acute and recovery phases from two KD patients by MS-based proteomic analysis

MS-based proteomic analysis is a powerful tool for discovering disease related proteins in sera. In order to obtain accurate information related only to KD, we compared the proteomes of paired sera collected during the acute and recovery phases from two KD patients (No. 3 and No. 6). For this purpose, we performed label-free relative quantitation analysis with MS data as described in the Materials and Methods section (Fig. S1). The patients were male, aged 2 and 5 years, with typical KD; they exhibited five or more of the clinical features of KD and had lower than normal body temperatures due to IVIG treatment (Table S1). By using MS analysis, we identified and used 65,514 peptides derived from 1,813 proteins for relative quantitative analysis (Table S2). Among these, we found that 391 peptides derived from 56 proteins were upregulated and 102 peptides derived from 40 proteins were downregulated during the acute phase of KD compared to its recovery phase. In order to narrow down the KD-related proteins among these, we selected proteins that yielded at least five peptides that were either up- or downregulated during the acute phase of KD. Consequently, 20 proteins that were upregulated and six that were downregulated proteins in the acute phase compared to the recovery phase were selected as KD-related proteins (Table 1). Among them, ceruloplasmin (CP), plasminogen (PLG), alpha-1-antichymotrypsin (SERPINA3), and complement system proteins (Complement C1r subcomponent (C1R), Complement factor B (CFB), Complement component 9 (C9), Complement C1s subcomponent (C1S), Complement component 4-A (C4A), Complement component 4-B (C4B), and Complement component 7 (C7)) have been frequently detected in the acute phase of several diseases. We also detected serum amyloid A-1 protein (SAA1), which was previously reported to be KD-related^12^. Therefore, our MS-based proteomic analysis indicates precise differences in protein expression levels in the serum between the acute and recovery phases of KD. Previous work identified transthyretin (TTR) as a potential marker for molecular diagnosis and progression monitoring of KD^11^. However, we did not detect TTR in this study, likely because it was removed by the immune depletion column prior to MS analysis.

### Validating candidate protein biomarkers for monitoring KD phases using western blotting

**Figure 1:**
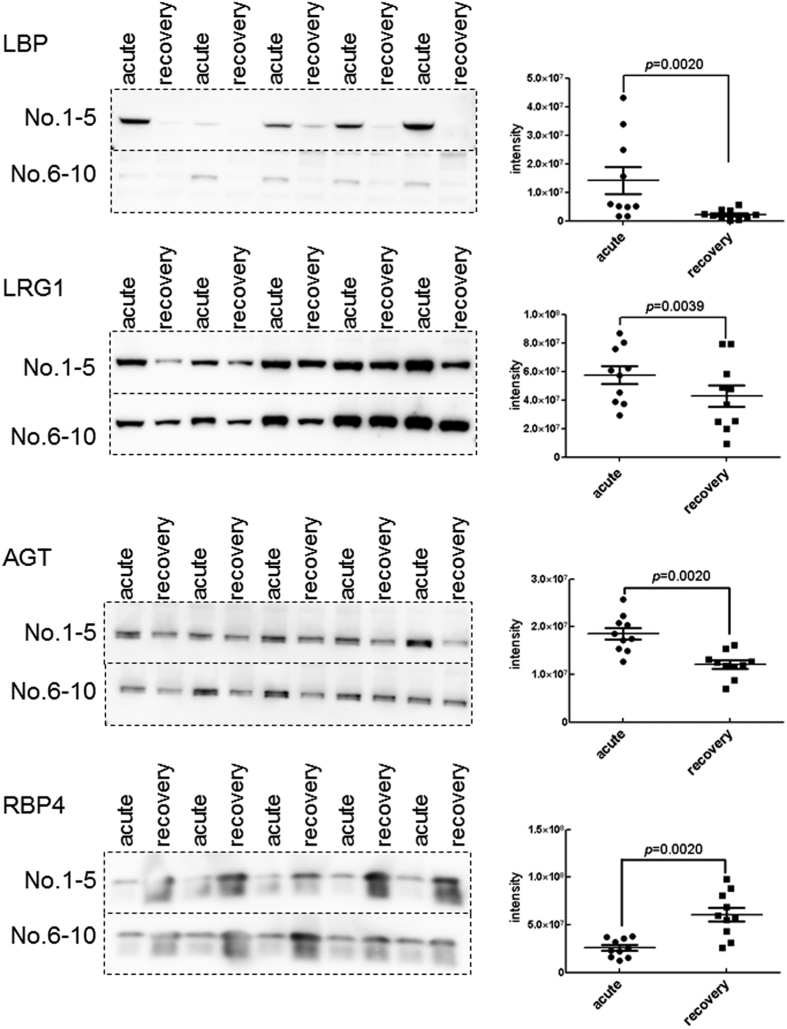
Validation of candidate protein biomarkers by western blots. Western blot analysis of LBP, LRG1, AGT, and RBP4 were performed. The paired sera (collected separately during the acute and recovery phases) obtained from ten KD patients (Patient No. 1 to 10) were used in this experiment. The panels on the right show the expression level of protein in the serum of each patient determined with the western blots shown on the left. The horizontal lines and I bars indicate the means and SEMs, respectively. *P* values are used to compare expression between the acute and recovery phases of KD. Each mean ± SEM is as following; LBP: 4.2E + 07 ± 2.6E + 07 (acute) and 1.6E + 07 ± 1.0E + 07 (recovery) (*p* < 0.0020), LRG1: 6.6E + 07 ± 2.1E + 07 (acute) and 5.1E + 07 ± 2.6E + 07 (recovery) (*p* < 0.0059), AGT: 2.7E + 07 ± 4.6E + 06 (acute) and 1.8E + 07 ± 3.9E + 06 (recovery) (*p* < 0.0020), and RBP4: 2.6E + 07 ± 9.3E + 06 (acute) and 6.1E + 07 ± 2.4E + 07 (recovery) (*p* < 0.002). The gels/blots were cropped and the full-length gels and blots were included in Supplementary Fig. 5.

To experimentally refine potential serum biomarkers for use in KD diagnosis, we performed western blots to monitor the expression levels of some KD-related proteins in paired sera containing high-abundance proteins obtained from ten KD patients during the acute and recovery phases (Figs 1 and S2). We determined that the expression levels of three proteins (LBP, LRG1, and AGT) were significantly higher in acute phase serum samples than in recovery phase samples. In contrast, the expression level of RBP4 was significantly lower in acute phase serum samples than in recovery phase samples. These observations are consistent with the results of our MS-based proteomic analysis.

### Validating potential protein biomarkers for monitoring KD phase using microarray enzyme-linked immunosorbent assays (ELISAs)

**Figure 2:**
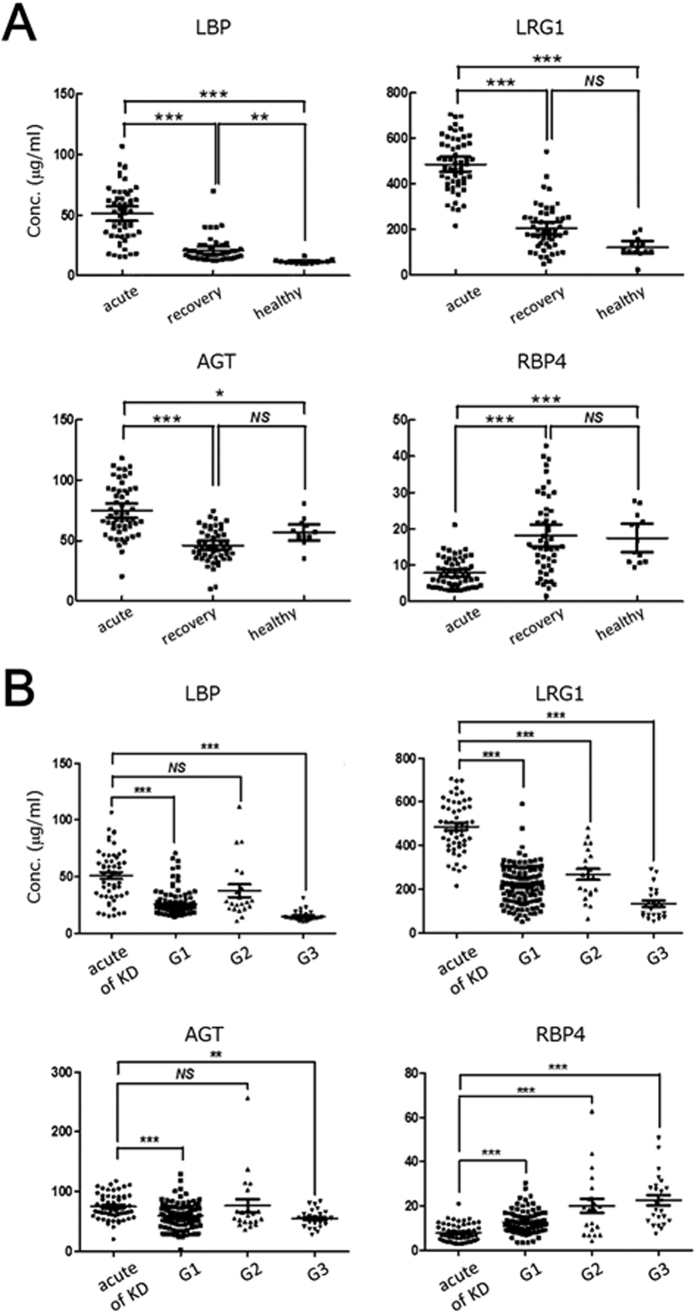
Validation of candidate protein biomarkers by microarray ELISAs. Panel A shows that expression levels of the four candidate biomarkers were significantly different in the acute phase of KD compared to the recovery phase of KD patients or healthy subjects. Panel B shows that the expression levels were significantly different in patients during the acute phase of KD compared to patients with other illnesses (G1: viral infections, G2: bacterial infections, and G3: autoimmune disease in childhood), except for LBP and AGT in G2. Microarray ELISAs were performed for LBP, LRG1, AGT, and RBP4, as described in the Materials and Methods section. The sera used in this experiment are described in Tables S1 and S3. The horizontal lines and I bars indicate means and the SEMs, respectively. *P* values are used for the comparison of the expression level among the acute and recovery phases of KD and other illnesses; ***p < 0.001, **p < 0.01, *p < 0.1, NS: non-significant. Each mean ± SD is described in Table 2.

**Figure 3:**
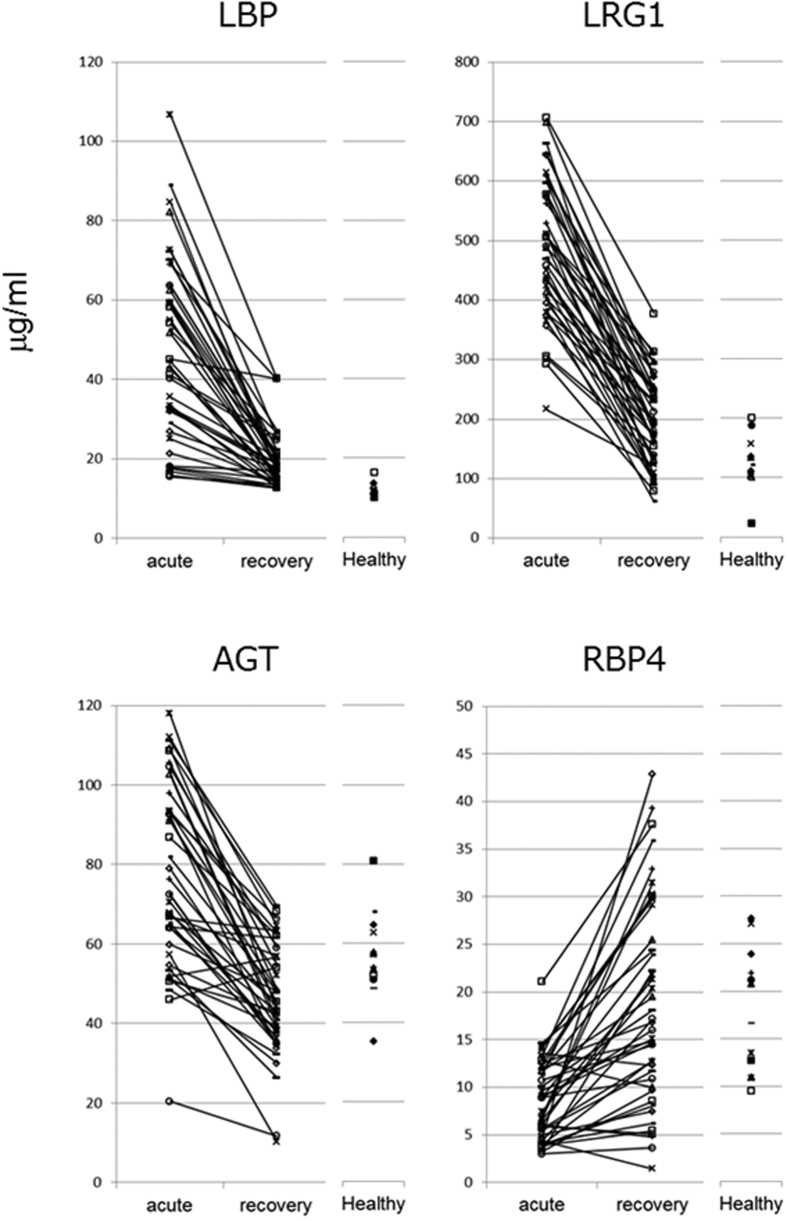
Changes in protein levels between acute and recovery phase sera from KD patients measured by microarray ELISA. Expression levels of each protein in the paired sera obtained during the acute and recovery phases from 42 KD patients and in the sera from 13 healthy subjects as measured by microarray ELISA. The solid line indicates the paired sera from each patient.

In order to carry out further experimental validation, we determined the expression levels of four proteins in the sera of KD patients and healthy subjects by microarray ELISAs, without depleting high-abundance proteins (Fig. 2A). Even when we examined a large number of patient sera, the concentrations of three proteins (LBP, LRG1, and AGT) were significantly higher in the acute phase than the recovery phase (*p* < 0.001), while the concentration of RBP4 was significantly lower in the acute phase compared to the recovery phase *(p* < 0.001) (Fig. 2, Table 2). In addition, receiver operating characteristic (ROC) curve analysis indicated that these KD-related proteins were useful for monitoring KD phase (Fig. S3). In particular, we observed that LBP and LRG1 expression levels in paired sera obtained during the acute and recovery phases from 42 KD patients decreased when their fever subsided (Fig. 3).

### Verification of candidate biomarkers for KD diagnosis

In order to evaluate the potential of the four KD-related proteins for use in distinguishing between KD and other childhood illnesses such as viral infections (G1), bacterial infections (G2), and autoimmune diseases (G3), we determined their expression levels in sera from patients suffering either from KD or these other illnesses using microarray ELISAs. The concentrations of three proteins (LBP, LRG1, and AGT) were significantly higher (*p* < 0.001) in the sera from the acute phase of KD than the other illnesses, except for LBP and AGT in G2. The concentration of RBP4 was significantly lower in the serum of acute phase of KD than the other illnesses (Fig. 2B). RBP4 was previously reported to increase after IVIG therapy in the serum of KD patients^11^. In this study, we found that the serum level of RBP4 was lower in acute phase than in recovery phase (two samples) of KD patients not subjected to IVIG therapy, or in samples from healthy subjects or those with other illnesses.

**Figure 4:**
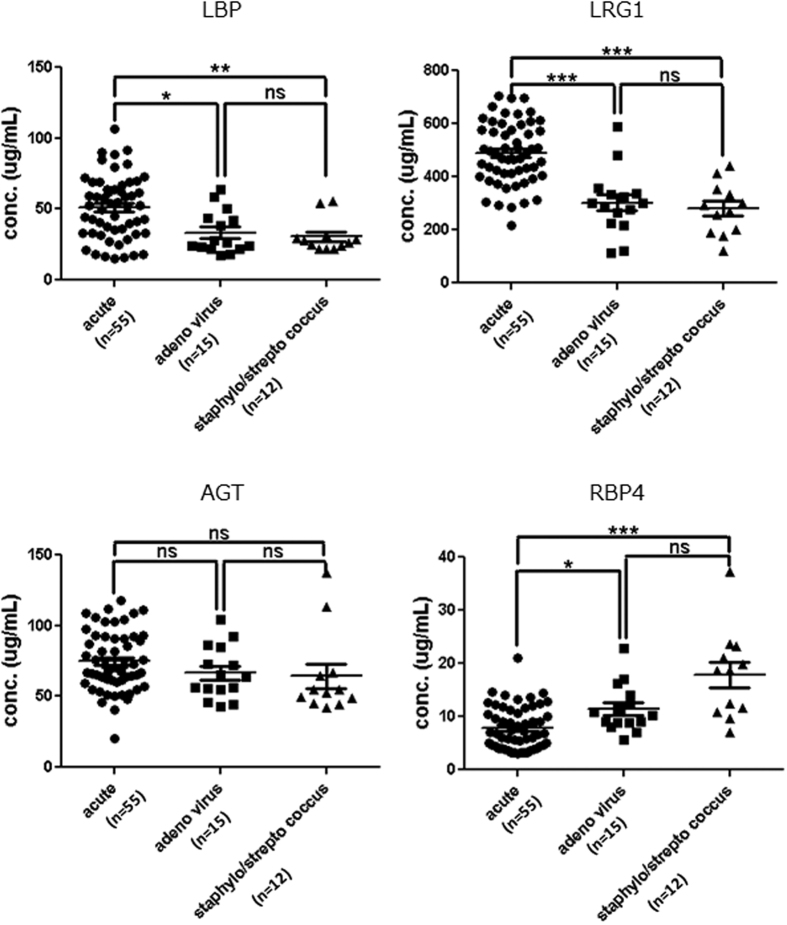
Verification of candidate proteins for KD diagnostic biomarkers by microarray ELISAs. Expression levels of the four candidate biomarkers were evaluated in the acute phase of KD and other infections that mimic KD, such as human adenovirus, *Staphylococcus*, and *Streptococcus* species. The serum concentrations of three proteins (LBP, LRG1, and RBP4) differed significantly in the acute phase of KD in comparison with these infections. The sera used in this experiment are described in Tables S1 and S3. The horizontal lines and error bars indicate means and SEMs, respectively. *P* values were used to evaluate the significance of differences between the acute phase of KD and these infections: ***p < 0.001; **p < 0.01; *p < 0.1; ns: non-significant.

Morbidity of KD is high in 0–5 year-olds. Therefore, we evaluated the usefulness of these biomarkers in patients under 5 years of age. The conclusion of the ROC curve analysis was consistent with the results obtained in children aged from 0 to 5 years, comprising almost all typical KD patients (Fig. S4). Therefore, these results suggest that these proteins are potential serum biomarkers during the acute phase of KD. In addition, some diseases caused by adenovirus*, Staphylococcus*, and *Streptococcus* species infection mimic KD in clinical settings^13^. Therefore, we next evaluated whether these KD-related proteins were useful for distinguishing KD from these diseases. The concentrations of three proteins (LBP, LRG1, and RBP4) significantly changed in sera from the acute phase of KD, but not in the other diseases (Fig. 4). These results suggested that LRG1 should be a particularly effective serum biomarker for distinguishing KD from these illnesses.

## Discussion

KD is characterized by acute systemic vasculitis in childhood. In clinical settings, early diagnosis of KD is crucial for preventing cardiovascular complications such as CALs. Because existing clinical criteria for KD are often insufficient for early and definitive diagnosis, biomarkers can help diagnosis of the disease and aid selection of the best treatment. Therefore, diagnosis using KD-related proteins will facilitate early and uniform diagnosis that is not reliant on the subjectivity and experience of physicians solely using clinical criteria.

Recently, many genetic studies of KD patients have been performed that found associations between genetic polymorphisms with susceptibility to KD and development of CALs^2^. Genes encoding inositol 1,4,5- three phosphorus acid 3-kinase C (*ITPKC*)^14^, caspase-3 (*CASP3*)^15^, B lymphocyte kinase (*BLK*), human leukocyte antigen (*HLA*), CD40 (*CD40*), and Fc fragment of Immunoglobulin G (IgG) low-affinity IIa receptor (*FC-GR2A*)^16^ were identified as products of KD susceptibility genes^17^. However, these genetic characteristics have not been studied sufficiently to understand their impact on KD pathogenesis and diagnosis. Therefore, it is difficult to diagnose patients with KD definitively by analyzing KD-susceptibility gene expression. In addition, no genetic biomarkers are available for promptly verifying a KD diagnosis in clinical settings. On the other hand, Kusuda *et al*. discovered KD-specific molecules that are linked to microbe-associated molecular patterns (MAMPs) derived from biofilms and that decrease in level following IVIG treatment, in the serum of KD patients^18^. It can be difficult, however, to distinguish between KD and bacterial infections solely by analyzing these molecules in clinical settings.

In contrast, serum biomarkers have the potential for wide clinical application through the development of rapid diagnostic techniques such as microarray ELISA. Some serum biomarkers are already used for clinical diagnosis of many diseases^19^. As for KD, blood vessel wall growth factor (VEGF)^20^,^21^ and N-terminal B-type natriuretic peptide (NT-proBNP) have previously been reported as candidate biomarkers^17^,^22^. However, these proteins still cannot be used as biomarkers for KD diagnosis in clinical settings. Clinical study results could not demonstrate adequate effectiveness.

MS-based proteomic analysis is often used for biomarker discovery. Although protein biomarkers are usually present at low concentrations in serum^23^, certain highly abundant serum proteins can obscure the presence of many other less abundant ones. A vast dynamic range of protein abundance, up to 12 orders of magnitude, complicates the identification of candidate biomarkers in the serum proteome using proteomics approaches^23^,^24^. Therefore, removing such highly abundant proteins is an essential first step when analyzing the serum proteome by liquid chromatography (LC)-MS/MS for biomarker discovery in order to reduce sample complexity^25^. Therefore, many prefractionation methods have been developed for biomarker discovery by shotgun LC-MS/MS^26^. In this study, we carried out prefractionation of the serum proteome using high performance liquid chromatography (HPLC) with a C4 reversed-phase column. We were able to achieve highly reproducible fractionation in this manner, and identified numerous peptides derived from many proteins at various concentrations in serum as being available for relative quantitation (Table S2). Consequently, we could search for KD-related candidates from among these numerous serum-derived peptides.

For effective biomarker discovery, it is also important to collect a good sample. Environmental factors and genetic backgrounds may affect the serum proteome, indicating that disease-specific factors are difficult to identify by comparison between different individuals. Therefore, KD-specific biomarkers for use in clinical settings have not been established in previous proteomic studies using many serum samples. To screen for KD-related proteins, in this study we obtained paired sera from two KD patients during the acute and recovery phases and subjected them to shotgun LC-MS/MS. Although the number of patients was small, we simply compared serum proteome from the same patient during both the acute and recovery phases, without needing to consider differences between two individuals. We could expect to obtain important information on KD-related proteins at the beginning of this study.

In this manner, we successfully obtained indications as to the identity of KD-related proteins for use in effective monitoring of KD phase. Based on these indications, we validated experimentally the differentially expressed proteins using several clinical samples and verified that these proteins are actually associated with KD. We thereby effectively demonstrated without wasting time, effort, or expense that, among these proteins, four in particular may be potential serum biomarkers for KD following experimental validation using microarray ELISAs with specific antibodies.

In particular, we determined that LBP and LRG1 are present at very high levels in serum from KD patients in the acute phase compared with both patients in the recovery phase and with healthy subjects. The blood levels of these proteins are high enough to detect precisely and quickly using microarray ELISAs. LBP and LRG1 may therefore be useful biomarkers for monitoring KD phase in clinical settings, although the availability of clinical samples is often limited. Additionally, RPB4 represents a promising potential biomarker for monitoring disease and therapeutic efficacy^11^.

Additionally, KD pathogenesis is probably mediated by various molecules. Therefore, an assay using multiple biomarkers with different specificities is more likely to yield a more definitive diagnosis than a single biomarker assay. Several potential serum biomarkers have already been developed for KD diagnosis^17^. Although these markers can reveal the intensity of inflammation in KD, it remains difficult to distinguish this condition from other diseases in clinical settings. By contrast, the expression pattern of each KD-related protein identified in this study may be of diagnostic value in distinguishing KD from other childhood diseases that mimic KD. Accordingly, these proteins should be validated within a prospective cohort including various childhood febrile diseases with similar or overlapping symptoms. Further large-scale studies will be necessary to confirm the diagnostic utility of these serum protein biomarkers and support this finding.

It is possible that these proteins may contribute to KD pathogenesis, although the relation between these proteins and KD remains to be elucidated. LBP plays an important role in the innate immune response to bacteria and fungi^27^. LBP transmits a signal within a cell through a receptor, CD14, on the cell membrane of phagocytic cells such as macrophages, and promotes the secretion of various inflammatory cytokines^28^. In fact, we observed high concentrations of LBP in serum derived from some patients with bacterial infections (G2) (Fig. 2B). The serum concentrations of inflammatory cytokines, such as TNF-α, interleukin 1, and interleukin 6, were remarkably high in KD patients compared to healthy subjects^29^,^30^. The pathogenesis of KD can be summarized as abnormal activation of the immune system. Therefore, this may indicate that bacterial infections followed by LBP upregulation contribute in part to the onset of KD. LRG1 is associated with fibrosis and blood vessel remodeling^31^. LRG1 has also been reported to be an inflammatory biomarker in autoimmune diseases^32^,^33^. Rheumatoid arthritis (RA) is an inflammatory disorder and an autoimmune disease, in which the body’s immune system mistakenly attacks the joints. LRG1 concentration was significantly elevated in the serum of RA patients compared to that of healthy subjects but decreased following anti-TNF therapy involving infliximab and etanercept^32^. Serum LRG1 concentration decreased significantly in all KD patients due to recovery in this study (Fig. 3), although anti-TNF therapy also has a beneficial effect on the systemic inflammatory process in KD^34^. These results suggest that the KD’s pathogenesis is abnormal activation of the immune system, like autoimmune disease. Therefore, these KD-related proteins may have potential use in elucidating KD pathogenesis, although further studies using cell models are needed.

## Materials and Methods

### Patients

We collected serum samples from patients admitted to the following hospitals in Japan: Yokohama City University Hospital and Medical Center, Kanagawa Children’s Medical Center, Showa General Hospital, Kobe Children’s Primary Emergency Medical Center, and Japanese Red Cross Wakayama Medical Center. Informed consent from either the patients or their guardians was obtained before blood samples were collected (Tables S1 and S3). In addition, the protocol of this survey and research plan has been approved by the Clinical Ethics Committee of Yokohama City University Medical Center (D1502021). This study was also performed with the approval of the Ethics Committee in each of the medical facilities. Informed consent was obtained from all patients and/or their guardians.

The subjects of this study were 64 patients with KD aged 0–12 years (55 serum samples were obtained in the acute phase and 51 in the recovery phase) (Table S1). KD diagnosis was performed according to clinical criteria^2^. The acute phase is defined as the febrile period before intervention. The recovery phase is defined as the afebrile period following intervention. 151 patients with other illnesses aged from 0 to 18 years, who were divided into group 1 (G1; viral infection), group 2 (G2; bacterial infection), and group 3 (G3; autoimmune disease), and thirteen healthy subjects aged 0–10 years were served as a control group (Table S3).

### Identification of candidate protein biomarkers

In order to identify KD-related proteins, we performed shotgun LC-MS/MS followed by label-free relative quantitation of peptides in sera obtained from two patients with KD during the acute and recovery phases. A schematic overview of our MS-based proteomic analysis workflow is summarized in Fig. S1.

### Sample preparation for MS analysis

We used a Human 14 Multiple Affinity Removal System (MARS) column (Agilent Technologies, Palo Alto, CA, USA) to remove 14 human proteins (albumin, IgG, antitrypsin, Immunoglobulin A (IgA), transferrin, haptoglobin, fibrinogen, alpha2-macroglobulin, alpha1-acid glycoprotein, IgM, apolipoprotein AI, apolipoprotein AII, complement C3, and transthyretin) that are highly abundant in serum. Each serum sample was diluted with buffer A (Agilent Technologies) for MARS column, and passed through a cellulose acetate spin filter (0.22 μm, Agilent Technologies). Employing a HPLC system, a Chromaster system (Hitachi, Tokyo Japan), the preparative serum sample was injected into a MARS column (10 × 100 mm), and the flow-through fractions were subsequently collected (Fig. S5). The proteins were concentrated by centrifugal ultrafiltration using a spin concentrator (5 K MWCO, 4 ml, Agilent Technologies) and the buffer was exchanged to 0.1% trifluoroacetic acid (TFA). Next, we carried out fractionation of the serum proteome using HPLC with a C4 reversed-phase column. After exchanging the buffer, each serum sample (400 μg) was injected into a Vydac C4 reversed phase column (4.6 × 250 mm, Grace Vydac, Columbia, MD, USA) on a Chromaster system. The HPLC separation conditions were as follows: solvent A, 0.1%(v/v) TFA; solvent B, 0.1%(v/v) TFA/100%(v/v) 2-propanol; flow-rate, 0.7 ml/min; gradient, 0–15 min (0% solvent B), 15–18 min (25% solvent B), 18–60 min (100% solvent B); detection wavelength, 280 nm; column oven temperature, 40 °C. Fractions were collected every 1 min from 24 to 44 min (20 fractions) after sample injection. Each fraction was divided into two equal aliquots that were lyophilized and stored at −80 °C until use. To prepare peptides for MS, each sample was dissolved in lysis buffer (2 M urea, 0.5 M thiourea, and 50 mM NH_4_HCO_3_), reduced with Tris (2-carboxyethyl) phosphine (TCEP) at a final concentration of 5 mM, and subsequently alkylated with methyl methanethiosulfonate (MMTS) at a final concentration of 20 mM. Protein solutions were subjected to proteolysis with bovine trypsin (final concentration, 15 ng/μl) (Promega, Madison, WI, USA) at 37 °C for 16 h. To stop hydrolysis, TFA was added to a final concentration of 1%(v/v), and precipitates were removed by centrifugation. Acidified hydrolysates were desalted using C18 StageTips with C18 Empore disks^35^ (3 M, St. Paul, MN, USA). Eluted peptides were completely lyophilized.

### LC-MS/MS for label-free relative quantitation and identification of proteins

LC-MS/MS analysis was performed on a TripleTOF 5600 (AB Sciex, Foster City, CA, USA) coupled with a DiNa-AP (KYA Technologies, Tokyo, Japan). A pre-column HiQ sil C18W-3 (500 μm id × 1mm, KYA Technologies) and a nanoscale HiQ sil C18W-3 (100 μm id × 10 cm, KYA Technologies) were used. Peptide samples from each fraction were loaded on a reversed phase column and resolved at a flow rate of 200 nL/min with an acetonitrile/0.1% (v/v) formic acid gradient. Peptides were separated using a 145 min gradient program consisting of 5–40% buffer B for 120 min (buffer A: 2% acetonitrile, 98% water, 0.1% formic acid; buffer B: 80% acetonitrile, 20% water, 0.1% formic acid). Full-scan mass spectra were measured from *m/z* 400 to 1250 in positive-ion electro/spray ionization mode on a TripleTOF 5600 mass spectrometer operated in data-dependent mode. Label-free relative quantitation was performed using Progenesis LC-MS (v4.1, Nonlinear Dynamics, Durham, NC, USA). To identify peptides, peak lists were created using Progenesis LC-MS. The peak lists were used to search against human protein sequences (20,233 sequences) in the UniProt Knowledgebase (UniProtKB/Swiss-Prot) database (version Jan 2013) using MASCOT (v2.4.1, Matrix Science, London, UK). The search parameters were as follows: semitrypsin digestion with two missed cleavages permitted; variable modifications: N-terminal carbamylation, β-methylthiolate of cysteine, and oxidation of methionine; peptide charge of 2+, 3+, 4+; peptide mass tolerance for MS data ± 0.05 Da; and fragment mass tolerance ± 0.1 Da. We used a 1% overall false discovery rate (FDR) as a cutoff value to export our results from the database search using the software MASCOT. In addition, peptides that yielded a peptide ion score of greater than or equal to 30, were used for relative quantitation. Moreover, peptides that were either upregulated or downregulated during the acute phase of KD were extracted using the following parameters in Progenesis LC-MS: maximum fold change ≥2, analysis of variance (ANOVA) *p* < 0.001, and the average of normalized abundance (higher condition in Progenesis LC-MS) ≥500.

### Experimental validation of candidate protein biomarkers

The expression levels of some KD-related proteins discovered by proteomic analysis in the serum were investigated using western blots and microarray ELISAs. For western blot analysis, the diluted sera collected from ten KD patients during the acute phase and the recovery phase (Patient No. 1 to 10) were separated on 12.5% polyacrylamide gels before being electroblotted on to polyvinylidene difluoride (PVDF) membranes. The membrane was incubated in Blocking One solution (Nacalai Tesque, Kyoto, Japan) to block nonspecific binding before being incubated in anti-LBP antibody (diluted 1:3000; GeneTex, San Antonio, TX, USA), anti-LRG1 antibody (diluted 1:5000; Abcam, Cambridge, UK), anti-AGT antibody (diluted 1:100, Immuno-Biological Laboratories, Gunma, Japan), and anti-RBP4 antibody (diluted 1:1000; Abcam). Primary antibody incubation was followed by incubation with horseradish peroxidase–conjugated secondary antibody (diluted 1:5000). Signals were visualized using the ECL western blot detection system (GE Healthcare UK, Buckinghamshire, UK). To detect labeled proteins, membranes were scanned on a LAS-4000mini imaging system (GE Healthcare, Little Chalfont, UK). Band intensities were quantitated using MultiGauge 3.0 software (Fujifilm, Tokyo, Japan).

For the microarray ELISAs, we analyzed a total of 270 serum samples. These samples were collected from 55 patients in the acute phase of KD, 51 in the recovery phase of KD, 151 with other illnesses, and 13 healthy subjects were analyzed (Tables 2, S1, and S3). In this study, the following ELISA kits were used: LRG1 ELISA kit (Immuno-Biological Laboratories), AGT ELISA kit (Immuno-Biological Laboratories), LBP ELISA kit (Hycult Biotech, Uden, Netherlands), and RBP4 ELISA kit (CycLex, Nagano, Japan). Sera were prepared and each assay procedure was performed according to each manufacturer’s instructions.

### Statistical analysis

Statistical analysis was performed using GraphPad Prism (version 5.04, GraphPad Software, La Jolla, CA, USA). We used a Wilcoxon matched-pairs signed rank test for statistical analysis of our western blot results. As for our microarray ELISA results, we analyzed the serum protein levels that we measured using a Kruskal-Wallis test, followed by Dunn’s multiple comparison tests to determine statistical significance. We also generated ROC curves and area under the ROC curves (AUC) using GraphPad Prism. Experimental data derived are expressed as mean ± the standard error of the mean (SEM).

All methods were carried out in accordance with relevant guidelines and regulations. All experimental protocols were approved by the Clinical Ethics Committee of Yokohama City University Medical Center.

## Additional Information

**How to cite this article:** Kimura, Y. *et al*. Identification of candidate diagnostic serum biomarkers for Kawasaki disease using proteomic analysis. *Sci. Rep.* **7**, 43732; doi: 10.1038/srep43732 (2017).

**Publisher's note:** Springer Nature remains neutral with regard to jurisdictional claims in published maps and institutional affiliations.

## Supplementary Material

Supplementary Figures and Table S1,S3

Supplemental Table 2

## Figures and Tables

**Table 1:** Upregulated and downregulated proteins in the acute phase of KD identified by label-free relative quantitation analysis with shotgun LC-MS/MS analysis.

Acc No.*	Gene name	Description	Peptides**
*Up-regulated*			
P00450	CP	Ceruloplasmin	38
P00736	C1R	Complement C1r subcomponent	5
P00747	PLG	Plasminogen	9
P00751	CFB	Complement factor B	37
P01011	SERPINA3	Alpha-1-antichymotrypsin	86
P01019	AGT	Angiotensinogen	6
P02748	C9	Complement component C9	8
P02750	LRG1	Leucine-rich alpha-2-glycoprotein	19
P04114	APOB	Apolipoprotein B-100	9
P05155	SERPING1	Plasma protease C1 inhibitor	8
P09871	C1S	Complement C1s subcomponent	12
P0C0L4	C4A	Complement C4-A	16
P0C0L5	C4B	Complement C4-B	12
P0DJI8	SAA1	Serum amyloid A-1 protein	6
P10643	C7	Complement component C7	6
P18428	LBP	Lipopolysaccharide-binding protein	26
P19320	VCAM1	Vascular cell adhesion protein 1	5
P25311	AZGP1	Zinc-alpha-2-glycoprotein	7
Q06033	ITIH3	Inter-alpha-trypsin inhibitor heavy chain H3	7
Q14624	ITIH4	Inter-alpha-trypsin inhibitor heavy chain H4	17
*Down-regulated*			
P02753	RBP4	Retinol-binding protein 4	6
P02765	AHSG	Alpha-2-HS-glycoprotein	13
P06396	GSN	Gelsolin	9
P06727	APOA4	Apolipoprotein A-IV	7
P19823	ITIH2	Inter-alpha-trypsin inhibitor heavy chain H2	8
P19827	ITIH1	Inter-alpha-trypsin inhibitor heavy chain H1	12

^*^Acc No.; Accession numbers of UniProtKB/Swiss-Prot database.

^**^The number of peptides that were extracted using the following parameters in Progenesis LC-MS: max fold change ≥2, ANOVA *p* < 0.001, and the average of normalized abundance (higher condition in Progenesis LC-MS) ≥500.

**Table 2:** Clinical and biological characteristics of patients and controls.

		KD		Group 1 (n = 106)	Group 2 (n = 21)	Group 3 (n = 24)	Healthy (n = 13)
		Acute (n = 55)	Recovery (n = 51)				
Age (range (median))		0–12 (2)	0–12 (2)	0–15 (2)	0–17 (7)	2–18 (10.5)	0–10 (4)
Male (%)		54.5	60.8	60.4	52.4	25.0	46.2
KD	days	2–10	5–32	—	—	—	—
Conc. (μg/ml)*	LBP (mean ± SD)	51.1 ± 22.2	21.0 ± 10.1	25.7 ± 11.3	37.6 ± 25.5	15.1 ± 4.9	11.6 ± 1.8
	LRG1 (mean ± SD)	487.3 ± 117.1	206.4 ± 99.4	217.6 ± 93.2	268.2 ± 115.1	134.1 ± 67.6	120.6 ± 44.8
	AGT (mean ± SD)	74.8 ± 21.3	45.9 ± 14.0	58.9 ± 21.9	76.1 ± 49.5	54.9 ± 15.1	56.8 ± 10.9
	RBP4 (mean ± SD)	7.9 ± 4.0	18.2 ± 10.6	12.7 ± 4.9	20.2 ± 14.5	22.6 ± 11.5	17.5 ± 6.6

^*^Concentrations of LBP, LRG1, AGT, and RBP4 in serum were determined by microarray ELISA.
