# Supplementary material for: Identification of candidate diagnostic serum biomarkers for Kawasaki disease using proteomic analysis
Source: Sci Rep. 2017 Mar 6;7:43732. doi: 10.1038/srep43732 (PMC5338030; doi:10.1038/srep43732)
Supplement: Supplementary Figures and Table S1,S3 [file srep43732-s1.pdf]

Identification of candidate diagnostic serum biomarkers for Kawasaki disease using proteomic analysis

Yayoi Kimura, Masakatsu Yanagimachi, Yoko Ino, Mao Aketagawa, Michie Matsuo, Akiko Okayama, Hiroyuki Shimizu, Kunihiro Oba, Ichiro Morioka, Tomoyuki Imagawa, Tetsuji Kaneko, Shumpei Yokota, Hisashi Hirano, Masaaki Mori

Figure S1. A schematic overview of the MS-based proteomic analysis workflow.
The procedure used for MS-based proteomic analysis is described in the Materials and Methods section.

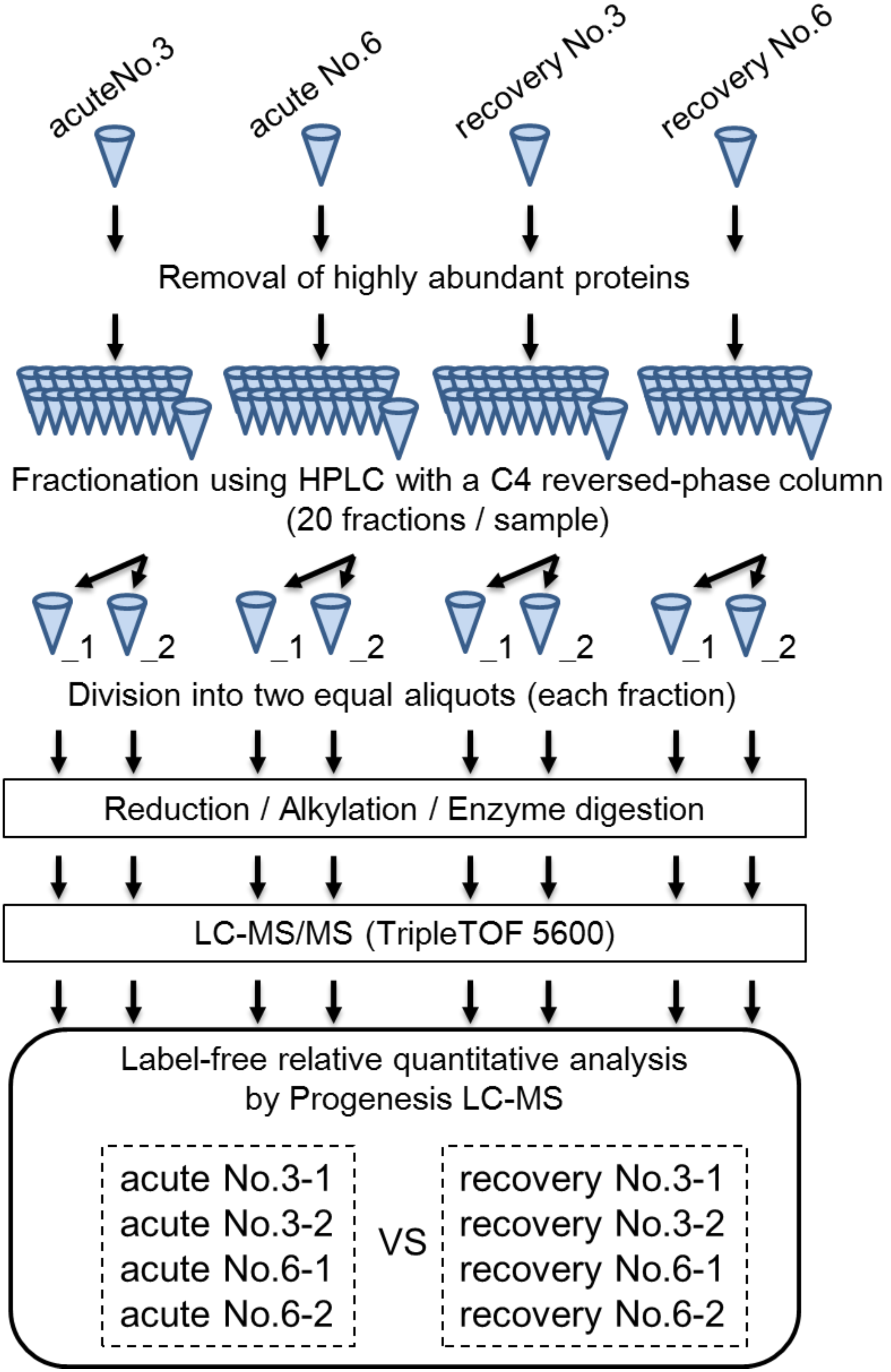

Figure S2. The full-length gels/blots of the cropped gels/blods in fig 1
The dotted lines indicate the cropped area for Figure 1.
M; molecular weight size marker

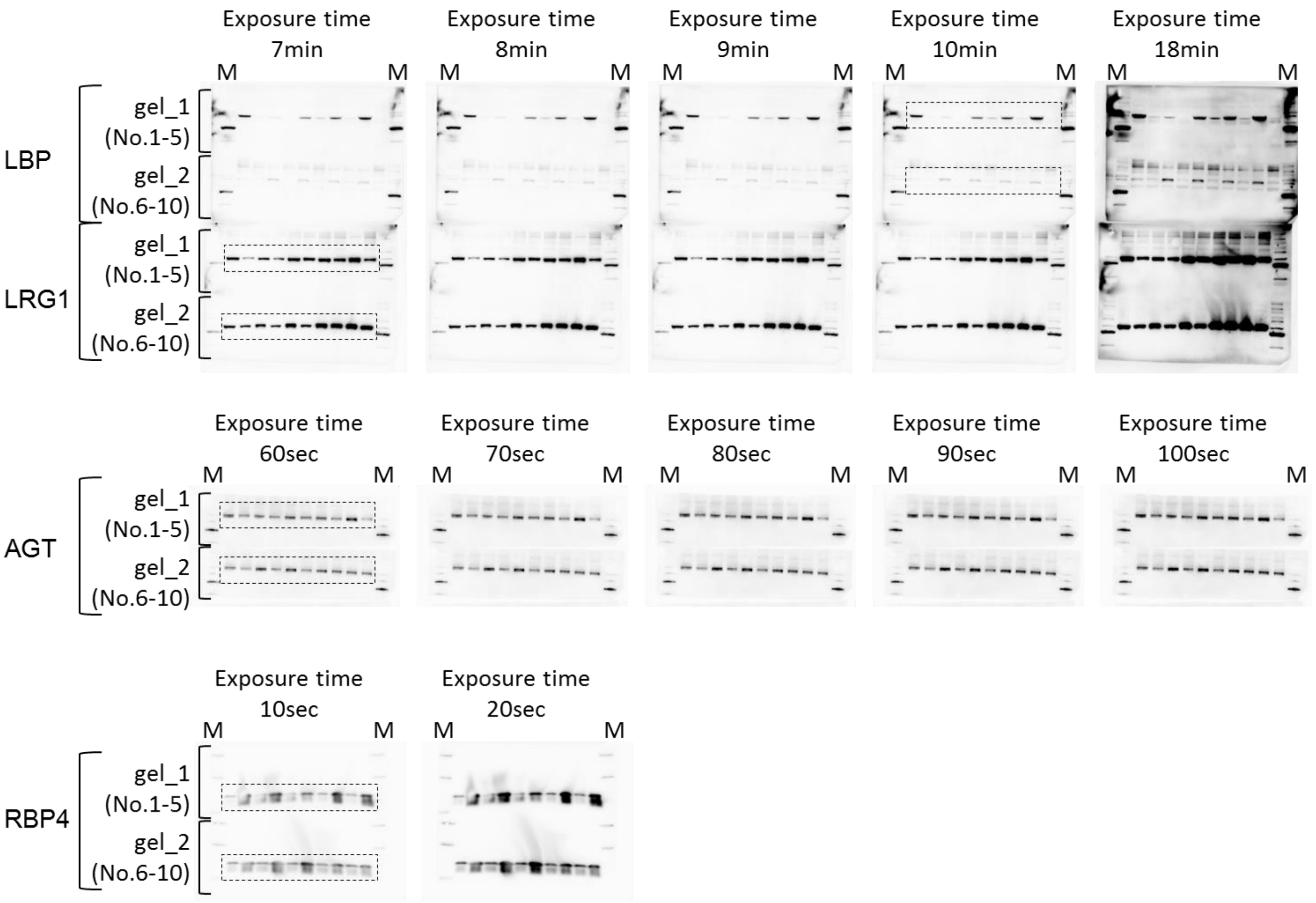

The dotted lines indicate the cropped area for Figure 1.
M; molecular weight size marker

Figure S3. Performance of candidate biomarkers in monitoring of the acute and recovery phase of KD

The diagnostic performance of those proteins was measured using ROC curve analysis for the acute phase of KD (55 sera) versus the recovery phase (51 sera).

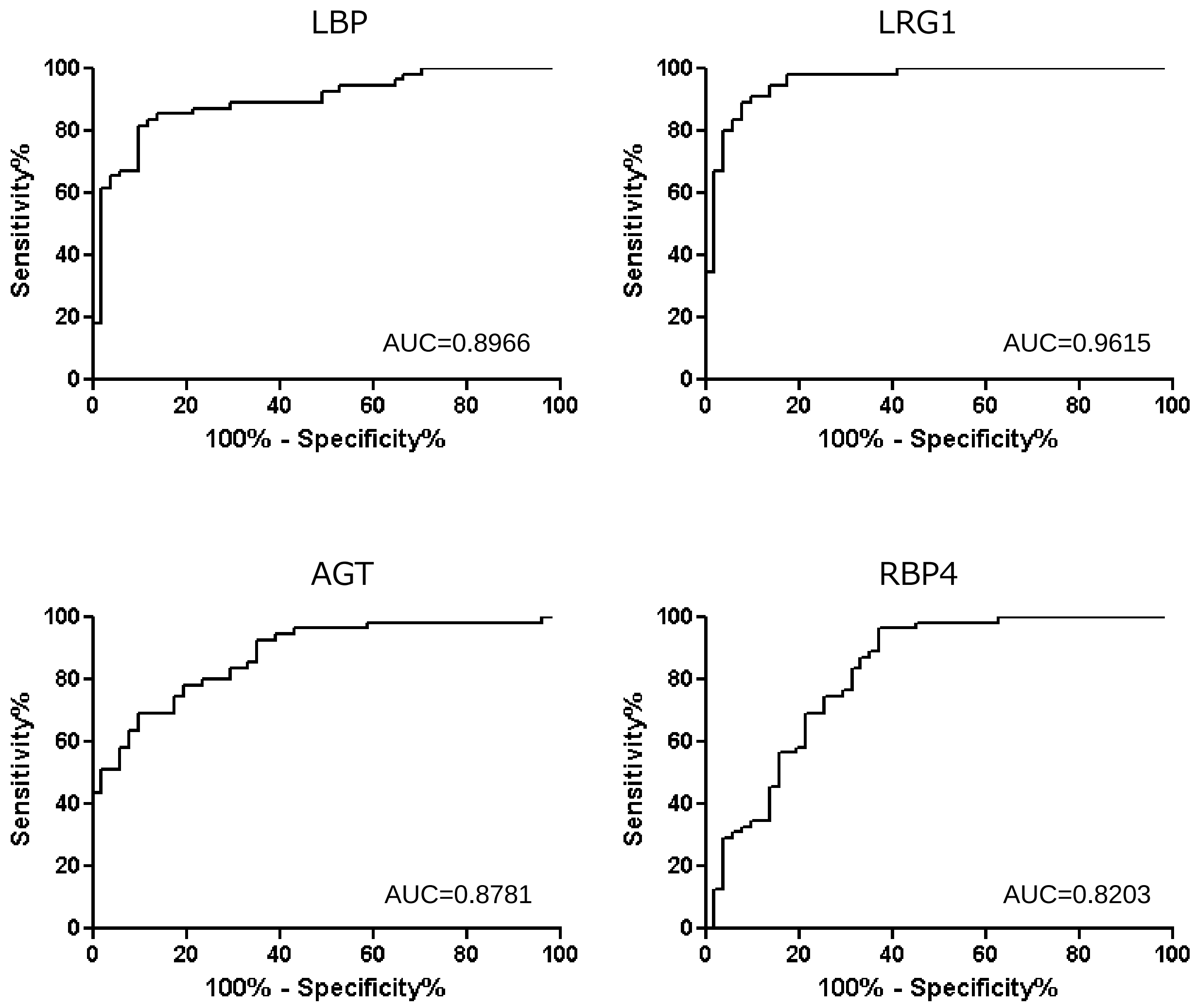

Figure S4. Diagnostic performance of candidate biomarkers in children under 5 years of age

The diagnostic performance of those proteins was measured using ROC curve analysis for the acute phase of KD (53 sera) versus the recovery phase of KD (50 sera) (a solid line) or control group (99 sera) (a dotted line).

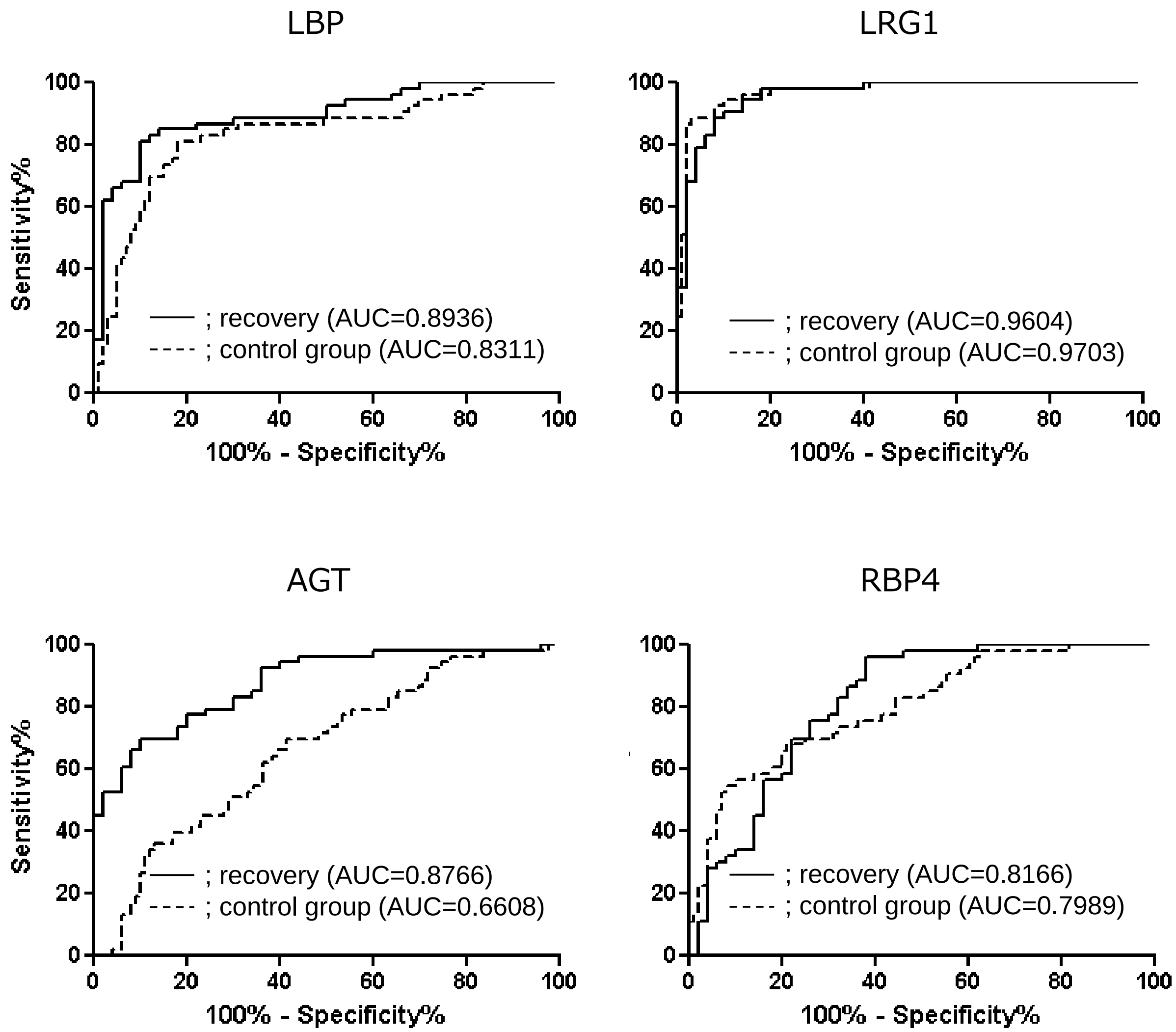

Figure S5. SDS-PAGE of crude and depleted serum samples.
The proteins were separated by 12.5% acrylamide gel. After electrophoresis, gel was stained by Oriole fluorescent gel stain. Asterisk indicates the typical high abundant proteins (*; albumin, **; IgG heavy chain, ***; IgG light chain).

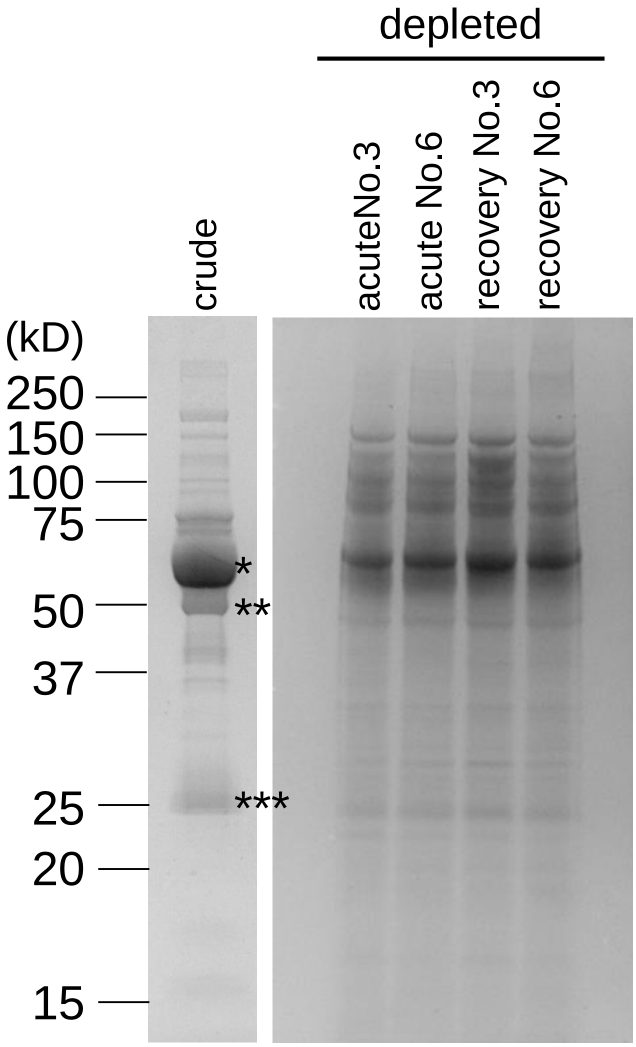

Table S1. Description of patients with KD.

	Patient No.	Days	Dose of IVIG(g/kg)	Age	Sex	MS-based analysis*	Western blot analysis*
				years			
acute	1	7	4	5	M		○
	2	10	4	5	F		○
	3	7	3	5	M	○	○
	4	8	4	2	M		○
	5	5	2	0	M		○
	6	8	4	2	M	○	○
	7	5	2	3	M		○
	8	5	2	3	M		○
	9	4	-	2	M		○
	10	4	2	2	M		○
	11	9	2	0	M		
	12	5	2	2	F		
	13	6	2	0	M		
	14	2	2	0	F		
	15	3	2	1	F		
	16	6	2	1	M		
	17	3	2	2	M		
	18	5	2	5	F		
	19	4	2	3	M		
	20	2	2	0	M		
	21	2	2	12	F		
	22	6	4	1	M		
	23	3	2	1	M		
	24	4	4	3	F		
	25	6	2	4	F		
	26	5	2	4	F		
	27	7	4	1	M		
	28	5	2	4	F		
	29	4	2	3	F		
	30	4	2	1	F		
	31	5	4	0	M		
	32	4	2	3	F		
	33	3	2	0	M		
	34	6	4	2	F		
	35	5	4	1	F		
	36	3	-	1	M		
	37	4	2	4	M		
	38	3	4	2	M		
	39	5	2	1	M		
	40	4	2	4	M		
	41	3	2	0	F		
	42	6	2	5	F		
	43	6	3	2	M		
	44	10	3	3	F		
	45	9	4	5	F		

	46	10	3	3	F		
	47	9	4	2	F		
	48	3	2	3	F		
	49	5	2	0	M		
	50	6	2	4	F		
	51	4	4	6	M		
	52	4	2	2	F		
	53	3	4	3	M		
	54	5	2	2	F		
	55	8	2	1	M		
recovery	1	16	4	5	M		○
	2	18	4	5	F		○
	3	13	3	5	M	○	○
	4	16	4	2	M		○
	5	16	2	0	M		○
	6	14	4	2	M	○	○
	7	25	2	3	M		○
	8	17	2	3	M		○
	9	8	-	2	M		○
	10	8	2	2	M		○
	11	12	2	0	M		
	12	9	2	2	F		
	13	12	2	0	M		
	14	5	2	0	F		
	15	7	2	1	F		
	16	12	2	1	M		
	17	8	2	2	M		
	18	9	2	5	F		
	19	7	2	3	M		
	20	7	2	0	M		
	21	7	2	12	F		
	22	15	4	1	M		
	23	7	2	1	M		
	24	8	4	3	F		
	25	9	2	4	F		
	26	9	2	4	F		
	27	13	4	1	M		
	28	9	2	4	F		
	29	7	2	3	F		
	30	10	2	1	F		
	31	14	4	0	M		
	32	12	2	3	F		
	33	9	2	0	M		
	34	10	4	2	F		
	35	9	4	1	F		
	36	31	-	1	M		
	37	8	2	4	M		
	38	9	4	2	M		

39	12	2	1	M
40	7	2	4	M
41	7	2	0	F
42	10	2	5	F
56	19	4	5	F
57	15	3	4	M
58	14	4	2	F
59	30	4	3	M
60	22	4	0	M
61	32	6	0	M
62	10	2	0	M
63	9	2	4	F
64	7	2	4	M

The asterisk indicates the serum samples of patients provided in each analysis.

Table S3. Description of patients with other illnesses and healthy subjects.

Group	Patient No.	Infection		Disease	Age	Sex
		Virus	Bacteria			
G1;	1	RS			1	F
Viral infection	2	Influenzae A			11	F
	3	Influenzae A			2	F
	4	Influenzae A			8	M
	5	Influenzae A			8	M
	6	Influenzae A			9	F
	7	Influenzae A			4	M
	8	Influenzae A			2	F
	9	Influenzae A			6	M
	10	Influenzae A			0	M
	11	Influenzae A			9	F
	12	Influenzae A			10	M
	13	Influenzae A			12	M
	14	Influenzae A			0	M
	15	Influenzae A			4	F
	16	Influenzae A			2	M
	17	Influenzae A			1	M
	18	Influenzae A			2	M
	19	Influenzae A			1	F
	20	Influenzae A			15	F
	21	Influenzae A			4	M
	22	Influenzae A			0	M
	23	Influenzae A			2	M
	24	Influenzae A			1	M
	25	Influenzae B			4	M
	26	Influenzae B			10	F
	27	Influenzae B			6	M
	28	Influenzae B			7	M
	29	Influenzae B			6	F
	30	Influenzae B			10	M
	31	Influenzae B			13	F
	32	Influenzae B			6	F
	33	Influenzae B			8	M
	34	Influenzae B			11	F
	35	Influenzae B			4	F
	36	Influenzae B			14	M
	37	Influenzae B			9	M
	38	Influenzae B			14	M
	39	Influenzae B			12	F
	40	Influenzae B			10	M
	41	Influenzae B			5	F
	42	Influenzae B			7	F
	43	Influenzae B			6	M
	44	Influenzae B			3	M
	45	Rota			0	M
	46	Rota			6	M

47	Rota	1	F
48	Rota	1	M
49	Rota	1	M
50	Rota	7	M
51	Rota	1	M
52	Rota	0	M
53	Rota	1	F
54	Rota	0	M
55	Rota	1	M
56	Rota	4	M
57	Rota	2	F
58	Rota	2	M
59	Rota	0	M
60	Rota	1	F
61	Rota	2	M
62	Rota	2	M
63	Rota	1	M
64	Rota	1	F
65	Noro	3	M
66	Noro	0	M
67	Noro	1	M
68	Noro	8	M
69	Noro	1	M
70	Noro	0	M
71	Noro	1	F
72	Adeno	2	F
73	Adeno	0	F
74	Adeno	1	M
75	Adeno	1	M
76	Adeno	3	M
77	Adeno	1	F
78	Adeno	7	F
79	Adeno	5	M
80	Adeno	6	M
81	Adeno	1	F
82	Adeno	1	M
83	Adeno	1	F
84	Adeno	1	F
85	Adeno	5	M
86	Adeno	5	M
87	RS	3	F
88	RS	0	M
89	RS	1	F
90	RS	0	F
91	RS	0	M
92	RS	0	F
93	RS	0	M
94	RS	0	M
95	RS	0	F

	96	RS			10	M
	97	RS			0	M
	98	RS			0	F
	99	RS			0	F
	100	RS			0	M
	101	RS			0	F
	102	RS			0	F
	103	RS			0	F
	104	RS			0	M
	105	RS			2	M
	106	RS			1	F
G2; Bacterial infection	107		Serratia marcescens		17	M
	108		Streptococcus pneumoniae		17	F
	109		Escherichia coli		8	F
	110		Clostridium difficile		7	F
	111		Clostridium difficile		15	F
	112		Streptococcus pyogenes		5	M
	113		Streptococcus pyogenes		4	F
	114		Streptococcus pyogenes		7	M
	115		Streptococcus pyogenes		9	F
	116		Streptococcus pyogenes		6	M
	117		Streptococcus pyogenes		10	M
	118		Streptococcus pyogenes		7	M
	119		Escherichia coli		0	M
	120		Escherichia coli		0	F
	121		Staphylococcus epidermidis		0	M
	122		Escherichia coli		0	M
	123		Staphylococcus aureus (MSSA)		11	F
	124		Staphylococcus aureus (MSSA)		7	F
	125		Micrococcus		14	M
	126		Stapyylococcus epidermidis (MSSE)		0	M
	127		Klebsiella		0	F
G3; Autoimmune disease	128			Idiopathic thrombocytopenic purpura (ITP)	2	M
	129			ITP	3	F
	130			ITP	4	M

	131	Juvenile idiopathic arthritis	18	F
	132	JIA	11	F
	133	Graft versus host disease (GVHD)	3	F
	134	Virus-associated hemophagocytic syndrome	2	M
	135	JIA	18	M
	136	JIA	12	F
	137	JIA	9	M
	138	JIA	9	F
	139	JIA	15	F
	140	JIA	12	F
	141	JIA	8	F
	142	JIA	7	F
	143	JIA	16	F
	144	JIA	14	F
	145	JIA	4	F
	146	JIA	13	F
	147	JIA	11	M
	148	JIA	10	F
	149	JIA	13	F
	150	JIA	5	F
	151	JIA	16	F
Healthy subject	152		4	F
	153		1	M
	154		1	M
	155		6	F
	156		7	F
	157		1	F
	158		5	F
	159		10	F
	160		6	M
	161		4	M
	162		0	M
	163		3	M
	164		6	F
